# Mineração de dados na avaliação de óbitos após cirurgia de amputação

**DOI:** 10.1590/1677-5449.008317

**Published:** 2018

**Authors:** Gabrielle dos Santos Leandro, Sheila Cristina Parolim, Claudia Maria Cabral Moro, Deborah Ribeiro Carvalho

**Affiliations:** 1 Pontifícia Universidade Católica do Paraná – PUCPR, Programa de Pós-Graduação em Tecnologia em Saúde – PPGTS, Curitiba, PR, Brasil.; 2 Prefeitura Municipal de Joinville, Secretaria Municipal de Saúde, Joinville, SC, Brasil.

**Keywords:** amputação, mortalidade, mineração de dados

## Abstract

**Contexto:**

A amputação e a desarticulação objetivam melhorar a saúde de um indivíduo, mas esses tratamentos apresentam taxas significantes de mortalidade que variam de acordo com os fatores relacionados.

**Objetivo:**

Identificar as associações entre os determinantes da mortalidade pós-operatória da amputação.

**Métodos:**

Estudo do tipo caso-controle (óbito *versus* não óbito) em que foi adotada a descoberta de regras de associação (abordagem da mineração de dados) e métricas epidemiológicas sobre 173 registros de pacientes amputados em um hospital público de Santa Catarina em 2014.

**Resultados:**

Os principais determinantes foram: idade > 60 anos [*odds ratio* (OR) = 3,0], sexo feminino (OR = 2,0), baixa escolaridade, hipertensão (OR = 3,0), diabetes (OR = 1,6) e tabagismo (OR = 1,8). Dos pacientes com idade entre 60 a 69 anos (38%), 87,9% evoluíram para alta, estando o óbito associado a doença vascular periférica. Quando a idade foi > 70 anos, embolia e trombose de artérias dos membros inferiores foram o fator de exceção (óbito). As patologias com maior associação ao óbito foram doença vascular (47,0%), diabetes (29,4%), doença cardíaca (razão de risco = 11,4), doença renal (OR = 10,4) e doença pulmonar (OR = 5,2). As cirurgias proximais estiveram mais associadas ao óbito do que as distais. Entre os pacientes que foram a óbito, 76,0% foram submetidos a raquianestesia e 24,0% a anestesia geral.

**Conclusão:**

A mineração de dados permitiu identificar as associações vinculadas ao óbito entre as diferentes variáveis e diagnósticos, como por exemplo, entre idade > 70 anos e diagnóstico de embolia e trombose de artérias dos membros inferiores.

## INTRODUÇÃO

 A amputação e a desarticulação são tratamentos com o intuito de melhorar a saúde de um indivíduo 1 . Porém, esses procedimentos apresentam elevadas taxas de mortalidade: 15 a 30,0% após 1 mês 2 ^-^ 4 , superior a 50,0% após 1 ano 3 , chegando até 77,0% após 5 anos 4 . Esses valores se diferenciam quando é considerada a localização da cirurgia, variando de 21,0% em amputação de nível transtibial até 38,0% em amputação de nível transfemural após 1 ano 5 . 

 A avaliação das taxas de mortalidade após um procedimento cirúrgico pode auxiliar na compreensão da situação de saúde de uma comunidade e na avaliação sobre o desempenho e a organização hospitalar, bem como a utilização dos recursos e a metodologia de trabalho 6 . Para tal, é importante identificar as relações existentes entre as características da amputação: causa, primária/recorrente, fonte de registro do óbito, entre outras. Uma forma de identificar essas relações é a partir de técnicas de mineração de dados (MD) 7 . 

 O objetivo deste estudo foi identificar as associações entre os determinantes da taxa de mortalidade pós-operatória de cirurgias de amputação adotando técnicas de MD. Os resultados deste estudo podem auxiliar na compreensão do risco de óbito pós-operatório da amputação e na identificação de seus principais determinantes, subsidiando o planejamento profilático e, consequentemente, aprimorando os serviços oferecidos pelo Sistema Único de Saúde. 

## MÉTODO

 Estudo do tipo caso-controle (óbito *versus* não óbito), retrospectivo, realizado por meio da revisão de registros eletrônicos de saúde (RES) de pacientes que sofreram amputação em um hospital público de Santa Catarina em 2014. O estudo obteve aprovação do Comitê de Ética em Pesquisa sob o parecer 1.669.393. 

 Foram analisados 173 RES, incluindo cirurgias de amputação e/ou desarticulação de coxa, hálux de dedo, pé e tarso, perna e membros inferiores, dedo e pododáctilos, dedo por dedo, antebraço, mão e punho, e pênis. 

 As variáveis utilizadas foram idade, sexo, estado civil, raça, escolaridade, profissão, hipótese diagnóstica, grupo da hipótese diagnóstica, diabetes, hipertensão, tabagismo, etilismo, saída (alta, transferência e óbito), reinternação, tempo de duração da cirurgia, tipo de cirurgia e tipo de anestesia. 

 Para a identificação dos determinantes, foram adotadas as três etapas previstas do processo *Knowledge Discovery in Databases*: pré-processamento, mineração de dados e pós-processamento 7 . Na primeira etapa, foram integrados dados sociais aos RES e calculada a taxa de mortalidade pós-operatória; na segunda, foram descobertas regras de associação a partir do algoritmo *Apriori* 8 ; e, na terceira, foi adotado o algoritmo de descoberta de regras de exceção 9 para identificação das situações de exceção. A regra de exceção pretende identificar, entre as regras descobertas, aquelas com mais chances de serem úteis ao especialista. Um exemplo de par de regras é mostrado a seguir: 

A → C (i%, j%) Regra do senso comum (alta cobertura e precisão)

 A,B → ¬ C (x%, y%) Regra de exceção (baixa cobertura e alta precisão) 7

 A primeira é a regra geral, seguida pela respectiva regra de exceção. 

 O símbolo “¬” significa negação lógica, a qual pode representar a simples alteração do valor possível da variável que se apresenta no consequente da regra 7 . Essas regras vêm acompanhadas de porcentagens, que são lidas da seguinte maneira: 

Regra: Se (i%) acontece em A, dos quais (j%) também apresentam C. 

Exceção: Se (x%) acontece em A associado a B, então (y%) acontece em C. 

 Essas regras são lidas da seguinte maneira, por exemplo, na Regra 1 no Quadro 1 : “27% dos pacientes têm idade entre 40 a 59 anos, dos quais 93,5% evoluíram para a alta”. Exceção: “1,7% dos pacientes têm idade entre 40 a 59 anos e possuem hipótese diagnóstica CID E115 (“Diabetes melito não insulinodependente - com complicações circulatórias periféricas” 10 ); desses, 66,7% evoluíram para o óbito”. 

**Quadro 1:** Regras e exceções.

**Regra geral**		**Exceção**
Idade		
1	SE idade entre 40 a 59 anos (27,0%) → Alta (93,5%)	SE idade entre 40 a 59 anos e CID E115 (1,7%) → ¬ Óbito (66,7%)
2	SE idade entre 60 e 69 anos (38,0%) → Alta (87,9%)	SE idade entre 60 e 69 anos e CID I739 (1,7%) → ¬ Óbito (66,7%)
3	SE idade 70 anos ou mais (35,0%) → Alta (86,9%)	SE idade é 70 anos ou mais e CID I743 (1,1%) → ¬ Óbito (100,0%)
Sexo		
4	SE feminino (31,2%) → Alta (85,2%)	SE feminino e CID E115 (1,1%) → ¬ Óbito (100,0%) SE feminino e CID I739 (1,7%) → ¬ Óbito (66,7%)
Estado civil		
5	SE viúvo (19,0%) → Alta (90,9%)	SE viúvo e aposentado (1,7%) → ¬ Óbito (66,7%)
6	SE casado (63,0%) → Alta (86,2%)	SE casado e CID I743 (1,7%) → ¬ Óbito (66,7%)
Raça		
7	SE raça branca (94,2%) → Alta (88,3%)	SE raça branca e CID I743 (1,7%) → ¬ Óbito (66,7%) SE raça branca e CID I739 (1,7%) → ¬ Óbito (66,7%)
Escolaridade		
8	SE alfabetizado (17,8%) → Alta (93,5%)	---
9	SE Ens. Fund. completo (13,8%) → Alta (87,5%)	---
10	SE Ens. Fund. incompleto (32,2%) → Alta (83,9%)	SE Ens. Fund. incompleto e CID I743 (1,1%) → ¬ Óbito (100,0%)
Hipertensão		
11	SE nega hipertensão (26,0%) → Alta (93,3%)	---
12	SE óbito (9,8%) → Hipertensão (88%)	---
13	SE óbito e hipertensão (8,7%) → Diabetes (86,7%)	---
Diabetes		
14	SE nega diabetes (24,0%) → Alta (87,8%)	---
15	SE óbito (9,8%) → Diabetes (82,0%)	---
16	SE diabetes (72,0%) → Alta (88,1%)	SE diabetes e CID I739 (1,1%) → Óbito (100,0%)
Tabagismo		
17	SE ex-tabagista (24,0%) → Alta (95,2%)	---
18	SE tabagista (21,0%) → Alta (83,8%)	---
19	SE nega tabagismo (27,0%) → Alta (95,7%)	---
Etilismo		
20	SE nega etilismo (46,0%) → Alta (92,4%)	SE nega etilismo e aposentado (1,7%) → Óbito (66,7%) SE nega etilismo e CID E115 (1,1%) → Óbito (100,0%) SE nega etilismo e CID I739 (1,1%) → Óbito (100,0%)
Reinternação		
21	SE mais de uma amputação (33,9%) → Alta (96,6%)	---
22	SE óbito (9,8%) → Uma amputação (88,2%)	---
23	SE óbito e Uma amputação (8,7%) → Raquianestesia (80,0%)	---
24	SE óbito e Uma amputação (8,7%) → Diabetes (80,0%)	---
25	SE uma amputação (65,5%) → Alta (85,1%)	SE uma amputação e CID E115 (2,9%) → Óbito (60,0%)
Tipo de cirurgia		
26	SE amp./des. perna e membros inferiores (13,0%) → Alta (90,9%)	---
27	SE óbito (9,8%) → Amputação de coxa (64,0%)	SE óbito e duração 0 a 30 min (1,7%) → Amp./des. de hálux de dedo (66,7%)
28	SE amputação de coxa (28,0%) → Alta (72,9%)	SE amputação de coxa e CID E115 (1,7%) → Óbito (100,0%) SE amputação de coxa e CID I743 (1,1%) → Óbito (100,0%)
Tipo de Anestesia		
29	SE óbito (9,8%) → Raquianestesia (76,0%)	SE óbito e duração de 0 a 30 min. (1,7%) → Anestesia geral (inalatória e venosa) (66,7%) SE óbito e duração de 151 a 180 min. (1,1%) → Anestesia geral (inalatória e venosa) (100,0%)
30	SE anestesia geral (inalatória e venosa) (13,0%) → Alta (82,6%)	---
Duração da cirurgia		
31	SE duração = 0 a 30 min. (6,9%) → Alta (75,0%)	SE duração = 0 a 30 min e anestesia geral (inalatória e venosa) (1,7%) → Óbito (66,7%)
32	SE duração = 61 a 90 min. (24,7%) → Alta (86,0%)	---
33	SE duração 91 a 120 min. (23,6%) → Alta (90,2%)	---
34	SE duração = 121 a 151 min. (12,1%) → Alta (85,7%)	SE duração = 121 a 151 min. e CID E115 (1,1%) → Óbito (100,0%)

O CID E115 corresponde a diabetes melito não insulinodependente – com complicações circulatórias periféricas. O CID I739 corresponde a doença vascular periférica não especificada. O CID I743 corresponde a embolia e trombose de artérias dos membros inferiores.

 Para complementar a análise dos dados, foram utilizadas as métricas epidemiológicas de associação: razão de risco (RR) e *odds ratio* (OR). 

## RESULTADOS

 Para melhor compreensão dos resultados, o número total de óbitos e amputações, RR e OR dos determinantes associados ao óbito, além de serem descritos nesta seção, são também apresentados na Tabela 1 . O mesmo foi feito com o conjunto de regras e exceções obtidas pelas técnicas de MD, que são exibidas no Quadro 1 . 

**Tabela 1:** Análise das variáveis sociodemográficas, morbidades, hábitos, grupo diagnóstico, localização da cirurgia e tipo de anestesia.

**Variáveis**		**Óbitos** **n**	**Não óbitos** **n**	**Total** **n**	**Razão de risco**	***Odds ratio***
Total		17	156	173	-	-
Idade	40 a 59 anos	2	45	47	Ref	Ref
	60 a 69 anos	8	58	66	2,8	3,1
	70 anos ou mais	7	53	60	2,7	3,0
Sexo	Feminino	8	46	54	2,0	2,1
	Masculino	9	110	119	Ref	Ref
Raça	Branca	17	146	163	-	-
	Parda	0	4	4	-	-
	Preta	0	4	4	-	-
	Não informado	0	2	2	-	-
Estado civil	Casado	13	96	109	2,2	2,0
	Solteiro	1	7	8	2,3	2,5
	Viúvo	3	30	33	Ref	Ref
	Divorciado	0	21	21	Ref	Ref
	Outros	0	1	1	Ref	Ref
	Não informado	0	1	1	-	-
Escolaridade	Ens. Fundamental incompleto	8	48	56	2,6	3,0
	Ens. Fundamental completo	2	22	24	1,5	1,6
	Analfabeto	0	6	6	Ref	Ref
	Alfabetizado	2	29	31	Ref	Ref
	Ens. Médio incompleto	0	1	1	Ref	Ref
	Ens. Médio completo	1	15	16	Ref	Ref
	Superior completo	0	1	1	Ref	Ref
	Não informado	4	34	38	-	-
	Diabético	14	111	125	1,5	1,6
	Não diabético	3	38	41	Ref	Ref
	Não informado	0	7	7	-	-
	Hipertenso	15	108	123	2,7	3,0
	Não hipertenso	2	43	45	Ref	Ref
	Não informado	0	5	5	-	-
	Tabagista e ex-tabagista	6	73	79	1,7	1,8
	Não tabagista	2	45	47	Ref	Ref
	Não informado	9	38	47	-	-
	Etilista, etilista-social e ex-etilista	2	41	43	0,6	0,6
	Não etilista	6	74	80	Ref	Ref
	Não informado	9	41	50	-	-
Diagnóstico	Doença cardíaca	1	0	1	11,4	-
	Doença pulmonar	1	2	3	3,8	5,2
	Doença renal	1	1	2	5,7	10,4
	Infecção	1	3	4	2,8	3,5
	Doença vascular	8	28	36	Ref	Ref
	Diabetes	5	81	86	Ref	Ref
	Ferida/úlcera	0	20	20	Ref	Ref
	Hanseníase	0	5	5	Ref	Ref
	Neoplasia	0	1	1	Ref	Ref
	Não informado	0	15	15	-	-
Localização	Em nível de antebraço	1	1	2	16,8	32,7
	Em nível de coxa	11	37	48	7,7	9,7
	Em nível de perna e membros inferiores	2	20	22	3,1	3,3
	Em nível de pé e tarso	1	27	28	Ref	Ref
	Hálux de dedo	2	48	50	Ref	Ref
	Dedo e pododáctilos	0	17	17	Ref	Ref
	Amputação de dedo por dedo	0	3	3	Ref	Ref
	Em nível de mão e punho	0	2	2	Ref	Ref
	Amputação de pênis	0	1	1	Ref	Ref
Anestesia	Anestesia geral (inalatória e venosa)	4	18	22	2,1	2,4
	Raquianestesia	13	108	121	Ref	Ref
	Anestesia local e sedação	0	10	10	Ref	Ref
	Raquianestesia e sedação	0	9	9	Ref	Ref
	Anestesia local	0	5	5	Ref	Ref
	Anestesia peridural	0	3	3	Ref	Ref
	Anestesia geral (venosa)	0	2	2	Ref	Ref
	Anestesia peridural e sedação	0	1	1	Ref	Ref

n indica o número de ocorrências e Ref indica que essas variáveis foram inclusas no grupo de referência para cálculo da razão de risco e do *odds ratio*.

 A taxa de mortalidade pós-operatória foi de 9,8%. Dos amputados, 73,0% eram idosos acima de 60 anos, totalizando 88,0% dos óbitos (OR = 3,0). Dos pacientes acima de 70 anos (35%), 86,9% evoluíram para a alta; contudo, todos os que tinham embolia e trombose de artérias dos membros inferiores (1,1%) foram a óbito. Dos pacientes com idade entre 60 e 69 anos (38,0%), 87,9% evoluíram para alta; mas, dos que tinham diagnóstico de doença vascular periférica não especificada (1,7% da amostra), 66,7% evoluíram para o óbito. Dos pacientes com idade entre 40 e 59 anos, 93,5% evoluíram para a alta; contudo, entre aqueles com diabetes melito não insulinodependente com complicações circulatórias periféricas (1,7% da amostra), 66,7% foram a óbito (Regras 3, 2 e 1 do Quadro 1 ). 

 A maioria dos amputados foram homens (68,8%); contudo, o risco e a chance de óbito foi duas vezes maior nas mulheres do que nos homens (RR = 2,0 e OR = 2,1). Das mulheres amputadas, 85,2% evoluíram para alta, e todas com diabetes melito não insulinodependente com complicações circulatórias periféricas (1,1%) foram a óbito ( Quadro 1 – Regra 4). 

 A raça branca esteve presente em 94,2% dos amputados e em todos os óbitos. Dos pacientes de raça branca, 88,3% evoluíram para alta; entretanto, na presença de embolia e trombose de artérias dos membros inferiores ou de doença vascular periférica não especificada (ambos com 1,7% da amostra), 66,7% foram a óbito ( Quadro 1 – Regra 7). 

 Tanto os casados como os solteiros apresentaram o dobro de chance de óbito se comparados aos divorciados e viúvos. Casados representaram 76,5% dos óbitos e 63,0% dos pacientes que fizeram cirurgia. Dos amputados casados, 86,2% evoluíram para alta; porém, naqueles com diagnóstico de embolia e trombose de artérias dos membros inferiores (1,7%), 66,7% foram a óbito ( Quadro 1 – Regra 6). 

 A baixa escolaridade foi predominante nos pacientes amputados: apenas 1,0% tinha ensino superior completo e 67,0% não cursaram o ensino médio. Dos 32,0% com ensino fundamental incompleto, 83,9% evoluíram para alta, e o óbito foi observado em todos quando associado ao diagnóstico de embolia e trombose de artérias dos membros inferiores (1,1%). Dos alfabetizados (17,8%), a alta aconteceu em 93,5%; e entre os pacientes com ensino fundamental completo (13,8%), a alta foi de 87,5% ( Quadro 1 – Regras 10, 8 e 9). Quanto à profissão, esta não foi informada em 70,0% dos registros, impossibilitando uma análise detalhada. Todavia, as profissões mais citadas foram: aposentado, do lar, motorista, pedreiro e vendedor. 

 A hipertensão foi encontrada em 71,0% dos amputados e em 88,0% dos óbitos. A chance de óbito foi três vezes maior nos pacientes hipertensos em comparação àqueles não hipertensos. Dos não hipertensos, 93,3% evoluíram para alta. A diabetes esteve presente em 72,0% dos amputados e em 82,0% dos óbitos. Dos amputados sem diabetes (24%), 87,8% ganharam alta. Dos diabéticos, 88,1% tiveram alta, mas todos os diabéticos com doença vascular periférica (1,1%) morreram. A diabetes esteve presente em 86,7% dos hipertensos que foram a óbito (8,7%) ( Quadro 1 – Regras 11, 14, 16 e 13). 

 Quanto ao uso do tabaco, 45,0% dos amputados faziam ou fizeram uso do tabaco, estando este associado a 35,0% dos óbitos; contudo, em 53,0% dos óbitos esse dado não foi informado. Dos amputados que negaram uso do tabaco (27,0%), 95,7% evoluíram para alta; dos 24,0% ex-tabagistas, 95,2% ganharam alta; já dos 21,0% tabagistas, 83,8% evoluíram para alta ( Quadro 1 – Regras 19, 17 e 18). Foi encontrado maior risco na seguinte ordem: tabagista, ex-tabagista e não tabagista. 

 Quanto ao etilismo, 25,0% faziam ou fizeram uso de bebida alcoólica, estando este associado a 12,0% dos óbitos; contudo, em 53,0% dos óbitos esse agravo não foi informado. Dos 46,0% que negaram etilismo, 92,4% evoluíram para alta. Entretanto, entre os que negaram etilismo e eram aposentados (1,7%), 66,7% evoluíram a óbito. Todos os amputados não etilistas com diagnóstico de diabetes melito não insulinodependente e com complicações circulatórias periféricas (1,1%) morreram, assim como os não etilistas com diagnóstico de doença vascular periférica (1,1%) ( Quadro 1 – Regra 20). 

 Dos que tiveram mais que uma amputação (33,9%), 96,6% evoluíram para alta, e 88,2% dos pacientes que foram a óbitos tiveram apenas uma amputação no ano. Dos pacientes que realizaram somente uma amputação no ano (65,5%), 85,1% evoluíram para alta. Dos 2,9% que realizaram uma amputação e tinham diabetes melito não insulinodependente com complicações circulatórias periféricas, 60,0% foram a óbito ( Quadro 1 – Regras 21, 22 e 25). 

 Na análise do grupo de patologias da hipótese diagnóstica, a doença vascular foi a principal causa de óbito (47,0%), seguida pela diabetes (29,4%). Contudo, o risco de óbito foi 11,4 vezes maior na presença da doença cardiovascular, cinco vezes maior na doença renal (RR = 5,7 e OR = 10,4), três vezes maior na doença pulmonar (RR = 3,8 e OR = 5,2), e duas vezes maior na infecção (RR = 2,8 e OR = 3,5) se comparado ao grupo de hipóteses diagnósticas doença vascular, diabetes, hanseníase, neoplasia e ferida/úlcera. 

 As cirurgias mais realizadas foram a de hálux de dedo (29,0%), de coxa (28,0%) e de pé e tarso (16,0%). Dos pacientes que evoluíram para óbito, 64,0% foram submetidos a amputações em nível de coxa, seguido por 12,0% de hálux de dedo e de perna, e membros inferiores respectivamente. O risco de óbito foi 16 vezes maior em cirurgias em nível de antebraço (RR = 16,8 e OR = 32,7) e sete vezes maior em nível de coxa (RR = 7,7 e OR = 9,7) se comparado ao das cirurgias em nível de pé, tarso, hálux, dedo e pododáctilos, dedo por dedo, mão e punho, e pênis. Na amputação de coxa, 72,9% dos pacientes ganharam alta, e o óbito esteve associado ao diabetes melito não insulinodependente com complicações circulatórias periféricas (1,7%) e a embolia e trombose de artérias dos membros inferiores (1,1%). Na amputação de perna e membros inferiores, 90,9% dos usuários evoluíram para alta ( Quadro 1 – Regras 28 e 26). 

 A anestesia mais usada foi a raquianestesia (70,0%) e a anestesia geral (inalatória e venosa) (13,0%). Dos pacientes que foram a óbito, 76,0% foram submetidos raquianestesia, sendo que 10,0% dos pacientes submetidos a raquianestesia morreram. O risco de óbito foi duas vezes maior quando foi usada a anestesia geral (inalatória e venosa) se comparada com as demais, estando presente em 24,0% dos óbitos. Dos óbitos com anestesia geral (66,7%), 1,7% tiveram duração da cirurgia entre 0 a 30 minutos. Quando a duração da cirurgia foi entre 151 a 180 minutos (1,1%) e a anestesia foi geral, todos morreram. Dos pacientes amputados com anestesia geral, 82,6% evoluíram para alta ( Quadro 1 – Regras 29 e 30). 

 Quanto à duração das cirurgias, a alta esteve presente em 75,0% das cirurgias com duração entre 0 a 30 min; mas quando essa duração esteve associada à anestesia geral (inalatória e venosa), o que foi observado em 1,7% da amostra, 66,7% dos pacientes morreram. As amputações com evolução para alta variaram da seguinte forma: entre 61 a 90 min (86,0% alta), de 91 a 120 min (90,2% alta), de 121 a 151 min (85,7% alta). Foi observado maior tempo de sobrevida na duração entre 91 a 120 min, diminuindo com o aumento e diminuição da duração desse período ( Quadro 1 – Regras 31, 32, 33 e 34). 

## DISCUSSÃO

 A taxa de mortalidade pós-operatória (9,8%) neste estudo foi baixa se comparada à de outros trabalhos que apresentaram taxas de 19,0% 4 . Alguns estudos trazem taxas de mortalidade global aos 30 dias entre 4,0% e 22,0% 4 ^,^ 5 ^,^ 11 . As diferenças nos valores podem ser explicadas por influências subjacentes dos serviços de saúde, decisões cirúrgicas e motivação dos pacientes por traz da decisão da amputação 4 . 

 Pacientes com idade acima de 60 anos tiveram três vezes mais chance de óbito do que os mais novos, sendo essa característica já observada em outros estudos 2 ^-^ 5 . Fortington et al. constatou que pessoas acima de 85 anos tiveram um tempo de sobrevida média de 8,8 meses após a amputação, enquanto que no grupo com pessoas mais novas a sobrevida foi de mais que 20 meses 4 . 

 As mulheres tiveram menos amputações, contudo morreram duas vezes mais se comparadas aos homens. No estudo de Rolim et al. aconteceu a mesma situação, em que 67,0% das amputações foram em homens e 33,0% em mulheres, apresentando mortalidade em 30 dias de 14,0% em homens e 15,0% em mulheres; em 90 dias, 22,0% em homens e 26,0% em mulheres; em 1 ano, 31,0% em homens e 36,0% em mulheres; e em 5 anos, 58,0% em homens e 61,0% em mulheres, mas essa diferença não foi citada em seu estudo 5 . 

 Fortington et al. citam que dos amputados a maioria eram homens (60,0%), com idade média de 72,1 anos, e as mulheres (40,0%) tinham idade média de 77 anos. A mortalidade em 30 dias foi de 28,0% em homens e 31,0% em mulheres; em 1 ano, 49,0% em homens e 52,0% em mulheres; e em 5 anos, 78,0% em homens e 80,0% em mulheres. Essa diferença é explicada pelas mulheres fazerem a amputação em idade mais avançada que os homens, sendo a idade um importante fator de risco 4 . 

 A mortalidade na raça branca foi predominante. Isso pôde ser justificado pelo fato de 89,7% da população de Santa Catarina ser da raça branca; além de que, no local do estudo, a cor branca é predominante devido ao fato da imigração alemã 12 . No estudo de Lavery et al., em que a raça predominante era a hispânica (78,1%), a taxa de sobrevida foi de 78,0% após 1 ano da amputação e 48,0% após 5 anos 13 . Situação semelhante foi encontrada em estudos em negros do Caribe e Barbados, 69,0% e 44,0%, respectivamente 14 , mas menor do que a relatada por índios norte-americanos 15 . 

 O estado civil casado foi predominante (76,5%), contudo as chances de óbito foram parecidas entre os casados (OR = 2,0) e solteiros (OR = 2,5) se comparados aos viúvos e divorciados. Essa característica foi atrelada ao perfil da população, pois outros estudos mostram que a maioria (58,0%) dos pacientes amputados moravam sozinhos e apenas 42,0% com algum cônjuge 4 . Essa informação denota os diferentes comportamentos em buscar saúde nos diversos tipos de estado civil, e que o planejamento do tratamento e da reabilitação deve considerar esse atributo. 

 A chance de óbito em pacientes hipertensos foi três vezes maior se comparada à dos não hipertensos, sendo que essa chance pode aumentar quando associados ao diabetes. Estudos relatam que pacientes com diabetes apresentaram mais amputações menores e duas vezes mais amputação em nível transtibial que amputação transfemural, diferentemente das pessoas que não tiveram diabetes 4 . 

 Quanto ao uso do tabaco, foi constatado risco na seguinte ordem: tabagista, ex-tabagista e não tabagista. Esse resultado condiz com estudo que afirma que, quanto ao óbito em 30 dias após a amputação, 33,0% fumavam e 25,0% não, em 1 ano 50,0% fumavam e 46,0% não, sendo em 5 anos 78,0% dos óbitos em tabagistas e 77,0% não 4 , confirmando que o uso do tabaco é um fator de risco para o paciente que realiza amputação. Contudo, tanto nessa variável como na do etilismo e na da profissão, foi constatado um grande percentual de registros incompletos e sem registros, o que dificulta uma análise eficiente. 

 As patologias mais frequentes no óbito pós-cirúrgico foram a doença vascular e diabetes, sendo o risco potencializado na presença da doença cardíaca, renal, pulmonar e infecções. Outro estudo traz que a etiologia predominante foi a doença vascular (doença arterial obstrutiva periférica – 87,0%) seguida pela infecção (7,0%), e que a presença de cardiopatia isquêmica e doença cerebrovascular teve impacto significativo como fator preditivo de menor sobrevida 5 . 

 Outro estudo mostrou que, dos óbitos após 30 dias da amputação, 45,0% tinham doença cerebrovascular, 33,0% doença renal, 32,0% doença cardíaca e 28,0% doença crônica pulmonar. Foi percebida probabilidade de morte de duas a três vezes maior em 30 dias para pessoas com doença cerebrovascular se comparada com as que não têm. Em pessoas com doença renal, notou-se probabilidade de morte 3,53 vezes mais em 1 ano e 5,35 vezes mais em 5 anos do que na ausência dessa morbidade 4 . A avaliação da reinternação fica prejudicada, tendo em vista a janela temporal adotada para a sistematização dos dados. 

 Em relação à localização da amputação, quanto mais invasiva a amputação, maior risco de óbito. Outro estudo aponta que as taxas de sobrevida aos 30, 90, 365 dias e 5 anos dos doentes submetidos a amputação menor foi de 95,0%, 91,0%, 79,0% e 55,0%, respectivamente. Já nos doentes submetidos a amputação maior as taxas foram de 82,0%, 70,0%, 62,0% e 35,0% respectivamente 4 . 

 Um desafio na cirurgia de amputação de membro inferior é decidir qual anestesia deve ser usada, sendo dada preferência às anestesias locais e à raquianestesia; contudo, existem poucas evidências que suportem a utilização de um tipo específico de anestesia devido ao perfil desses pacientes 16 ^,^ 17 , que sofrem um alto risco de eventos adversos 17 . Neste estudo, a raquianestesia esteve em 76,0% dos óbitos; contudo, mesmo que a anestesia geral (inalatória e venosa) estivesse presente em apenas 24,0% dos óbitos, pacientes que a fizeram tiveram 2,4 vezes mais chances de óbito do que as demais. 

 Porém, Chery et al. 16 afirma que as anestesias locais e/ou regionais para pacientes submetidos a amputação maior de extremidade estiveram associadas a uma menor incidência de complicações pulmonares pós-operatórias e arritmias cardíacas, devendo essa provavelmente ser o tipo de anestesia que favorece nesta situação. Moreira et al. 17 , que compararam a associação entre a raquianestesia e a anestesia geral nos pacientes que sofreram amputação de extremidade, concluíram que as taxas de mortalidade em 30 dias e de outras morbidades foram semelhantes em ambas anestesias, e que o tipo de anestesia não afetou significativamente a morbimortalidade. 

## CONCLUSÃO

 Os determinantes associados à mortalidade pós-operatória na amputação foram: idade acima de 60 anos (OR = 3,0), sexo feminino (OR = 2,0), raça branca, hipertensão (OR = 3,0), diabetes (OR = 1,6) e tabagismo (OR = 1,8). 

 As patologias que levaram mais ao óbito foram a doença vascular (47,0%) e a diabetes (29,4%). Foi observado risco de óbito aumentado na presença da doença cardiovascular (RR = 11,4), doença renal (OR = 10,4), doença pulmonar (OR = 5,2) e infecção (OR = 3,5). As cirurgias proximais estiveram mais associadas ao óbito do que as distais. As anestesias que mais se relacionaram ao óbito foram a raquianestesia e a anestesia geral (inalatória e venosa). 

 A MD permitiu identificar peculiaridades das associações entre os determinantes do óbito após a cirurgia de amputação. Por exemplo, as exceções que vinculam variáveis às diferentes hipóteses diagnósticas, aumentando as chances de óbito. 
