# Supplementary material for: Mineração de dados na avaliação de óbitos após cirurgia de amputação
Source: J Vasc Bras. 2018 Jan-Mar;17(1):10–8. [Article in Portuguese] doi: 10.1590/1677-5449.008317 (PMC5990270; doi:10.1590/1677-5449.008317)
Supplement: Translation

# Translation: Data mining to evaluate mortality after amputation surgery

**Authors:** Gabrielle dos Santos Leandro, Sheila Cristina Parolim, Claudia Maria Cabral Moro, Deborah Ribeiro Carvalho

**Affiliations:** 1 Pontifícia Universidade Católica do Paraná – PUCPR, Programa de Pós-Graduação em Tecnologia em Saúde – PPGTS, Curitiba, PR, Brasil.; 2 Prefeitura Municipal de Joinville, Secretaria Municipal de Saúde, Joinville, SC, Brasil.

**Keywords:** amputation, mortality, data mining

## Abstract

**Background:**

The objective of amputation and disarticulation is to improve health. However, these treatments are associated with significant mortality rates that vary in relation to risk factors.

**Objective:**

To identify associations between determinants of postoperative mortality after amputation surgery.

**Methods:**

Case-control study (death vs. no death) considering data from 173 patients who underwent amputation surgery at a public hospital in Santa Catarina state, Brazil. These data were analyzed using a data mining approach to discover association rules and epidemiologic association metrics.

**Results:**

The main determinants were age > 60 years (odds ratio (OR) = 3.0), female sex (OR = 2.0), low education, hypertension (OR = 3.0), diabetes (OR = 1.6), and smoking (OR = 1.8). Among patients aged 60-69 years, 87.9% survived to discharge from hospital. The exceptions occurred when patients in this age range had peripheral vascular disease. The same was true when age was > 70 years, among whom diagnoses of embolism and thrombosis of arteries of the lower extremities were the exception factors (associated with death). The most common pathologies associated with death were vascular disease (47.0%) and diabetes (29.4%), heart disease (relative risk = 11.4), renal disease (OR = 10.4), and lung disease (OR = 5.2). Proximal surgeries were more strongly associated with death than distal ones. Among the deaths, 76.0% had been given spinal anesthesia and 24.0% general anesthesia.

**Conclusion:**

Data mining enabled identification of associations between death and a variety of different variables and diagnostic hypotheses; for example, age > 70 years and diagnosis of embolism and thrombosis of arteries of the lower extremities.

## INTRODUCTION

 The objective of amputation and disarticulation is to improve the patient’s health. 1 However, these procedures are associated with elevated mortality rates: 15 to 30.0% after 1 month, 2 ^-^ 4 greater than 50.0% after 1 year, 3 and as high as 77.0% after 5 years. 4 The rates can be differentiated on the basis of site of surgery, varying from 21.0% for amputations at the transtibial level to 38.0% for amputation at the transfemoral level, at 1 year. 5

 Analysis of the mortality rates that follow a surgical procedure can improve understanding of the health status of a community and is important for assessment of the performance and organization of hospitals, use of resources, and working methodology. 6 In this case, it is important to identify relationships between amputation characteristics: cause, primary/recurrent, source of record of death, and other details. One way of identifying these relationships is by applying data mining (DM) techniques. 7

 The objective of this study was to identify associations between determinants of the postoperative mortality rate after amputation surgery, using DM techniques. The results of this study will improve understanding of the risk of postoperative death after amputation and help to identify its major determinants, providing a foundation for prophylactic planning and, consequently, for improvement of the services provided by the Brazilian National Health Service (SUS - Sistema Único de Saúde). 

## METHOD

 A retrospective case-control (death vs. no death) study was conducted by reviewing the electronic healthcare records (EHR) of patients who had had amputations at a public hospital in Santa Catarina in 2014. The study was approved by the Research Ethics Committee, under protocol number 1.669.393. 

 A total of 173 EHR were analyzed, covering operations for amputation and/or disarticulation at the thigh, hallux, foot, and tarsal, legs and lower limbs, fingers toes, finger by finger, forearm, hand and wrist, and penis. 

 The variables collected were age, sex, marital status, race, educational level, profession, diagnostic hypothesis, diagnostic hypothesis group, diabetes, hypertension, smoking, alcohol use, exit (discharge, transfer, or death), rehospitalization, duration of surgery, type of surgery, and type of anesthesia. 

 The method used for identification of determinants comprised the three steps of the Knowledge Discovery in Databases process: pre-processing, data mining, and post-processing. 7 The first step was to integrate the social data with the EHR and calculate the postoperative mortality rate; the second step was to use the *Apriori* 8 algorithm to discover the association rules, and the third was to use the algorithm for discovery of exception rules 9 to identify the exception scenarios. An exception rule is intended to identify, among the rules discovered, those that are most likely to be of use to the specialist. An example rule pair is shown below: 

A → C (i%, j%) Common sense rule (high coverage and high precision)

 A,B → ¬ C (x%, y%) Exception rule (low coverage and high precision) 7

The first is the general rule, followed by its respective exception rule.

 The “¬” symbol signifies logical negation, which could represent a simple alteration to the variable’s possible value that is the consequence of the rule. 7 These rules are generated with percentages, which are read in the following manner: 

Rule: If (i%) occurs in A, of which (j%) also exhibit C. 

Exception: If (x%) occurs in A associated with B, then (y%) occurs in C. 

 These rules are read in the following manner, for example, Rule 1 in Chart 1 : “27% of the patients are aged from 40 to 59 years, of whom 93.5% survived to discharge”. Exception: “1.7% of the patients are aged from 40 to 59 years and have diagnostic hypothesis ICD E115 (“Type 2 diabetes mellitus with circulatory complications”) 10 ; 66.7% of whom died”. 

**Chart 1:** Rules and exceptions.

**General rule**		**Exception**
Age		
1	IF age is 40 to 59 years (27.0%) → Discharge (93.5%)	IF age is 40 to 59 years and ICD E115 (1.7%) → ¬ Death (66.7%)
2	IF age is 60 to 69 years (38.0%) → Discharge (87.9%)	IF age is 60 to 69 years and ICD I739 (1.7%) → ¬ Death (66.7%)
3	IF age is 70 years or more (35.0%) → Discharge (86.9%)	IF age is 70 years or more and ICD I743 (1.1%) → ¬ Death (100.0%)
Sex		
4	IF sex is female (31.2%) → Discharge (85.2%)	IF sex is female and ICD E115 (1.1%) → ¬ Death (100.0%) IF sex is female and ICD I739 (1.7%) → ¬ Death (66.7%)
Marital status		
5	IF widowed (19.0%) → Discharge (90.9%)	IF widowed and retired (1.7%) → ¬ Death (66.7%)
6	IF married (63.0%) → Discharge (86.2%)	IF married and ICD I743 (1.7%) → ¬ Death (66.7%)
Race		
7	IF race is white (94.2%) → Discharge (88.3%)	IF race is white and ICD I743 (1.7%) → ¬ Death (66.7%) IF race is white and ICD I739 (1.7%) → ¬ Death (66.7%)
Educational level		
8	IF literate (17.8%) → Discharge (93.5%)	---
9	IF Primary Ed. completed (13.8%) → Discharge (87.5%)	---
10	IF Primary Ed. not completed (32.2%) → Discharge (83.9%)	IF Primary Ed. not completed and ICD I743 (1.1%) → ¬ Death (100.0%)
Hypertension		
11	IF no hypertension (26.0%) → Discharge (93.3%)	---
12	IF death (9.8%) → Hypertension (88%)	---
13	IF death and hypertension (8.7%) → Diabetes (86.7%)	---
Diabetes		
14	IF no diabetes (24.0%) → Discharge (87.8%)	---
15	IF death (9.8%) → Diabetes (82.0%)	---
16	IF diabetes (72.0%) → Discharge (88.1%)	IF diabetes and ICD I739 (1.1%) → Death (100.0%)
Smoking		
17	IF ex-smoker (24.0%) → Discharge (95.2%)	---
18	IF smoker (21.0%) → Discharge (83.8%)	---
19	IF non-smoker (27.0%) → Discharge (95.7%)	---
Alcoholism		
20	IF no alcoholism (46.0%) → Discharge (92.4%)	IF no alcoholism and retired (1.7%) → Death (66.7%) IF no alcoholism and ICD E115 (1.1%) → Death (100.0%) IF no alcoholism and ICD I739 (1.1%) → Death (100.0%)
Rehospitalization		
21	IF more than one amputation (33.9%) → Discharge (96.6%)	---
22	IF death (9.8%) → one amputation (88.2%)	---
23	IF death and one amputation (8.7%) → Spinal anesthesia (80.0%)	---
24	IF death and one amputation (8.7%) → Diabetes (80.0%)	---
25	IF one amputation (65.5%) → Discharge (85.1%)	IF one amputation and ICD E115 (2.9%) → Death (60.0%)
Type of surgery		
26	IF amp./disart. leg and lower limbs (13.0%) → Discharge (90.9%)	---
27	IF death (9.8%) → Amputation at thigh (64.0%)	IF death and duration 0 to 30 min (1.7%) → Amp./disart. of hallux (66.7%)
28	IF amputation at thigh (28.0%) → Discharge (72.9%)	IF amputation at thigh and ICD E115 (1.7%) → Death (100.0%) IF amputation at thigh and ICD I743 (1.1%) → Death (100.0%)
Type of Anesthesia		
29	IF death (9.8%) → Spinal anesthesia (76.0%)	IF death and duration 0 to 30 min. (1.7%) → General anesthesia (inhaled and venous) (66.7%) IF death and duration 151 to 180 min. (1.1%) → General anesthesia (inhaled and venous) (100.0%)
30	IF general anesthesia (inhaled and venous) (13.0%) → Discharge (82.6%)	---
Duration of surgery		
31	IF duration = 0 to 30 min. (6.9%) → Discharge (75.0%)	IF duration = 0 to 30 min and general anesthesia (inhaled and venous) (1.7%) → Death (66.7%)
32	IF duration = 61 to 90 min. (24.7%) → Discharge (86.0%)	---
33	IF duration 91 to 120 min. (23.6%) → Discharge (90.2%)	---
34	IF duration = 121 to 151 min. (12.1%) → Discharge (85.7%)	IF duration = 121 to 151 min. and ICD E115 (1.1%) → Death (100.0%)

ICD E115 is type 2 diabetes mellitus with circulatory complications. ICD I739 is peripheral vascular disease, unspecified. ICD I743 is embolism and thrombosis of arteries of the lower extremities.

 The epidemiological association metrics risk ratio (RR) and odds ratio (OR) were used to supplement the data analysis. 

## RESULTS

 To aid in interpretation of the results, total numbers of deaths and amputations, RR and OR for determinants associated with death will be described in this section and also presented in Table 1 . Similarly, the set of rules and exceptions derived using the DM techniques are also shown in Chart 1 . 

**Table 1:** Analysis of sociodemographic variables, morbidities, habits, diagnosis groups, surgery sites, and types of anesthesia.

**Variables**		**Death** **n**	**No death** **n**	**Total** **n**	**Risk ratio**	**Odds ratio**
Total		17	156	173	-	-
Age	40 to 59 years	2	45	47	Ref	Ref
	60 to 69 years	8	58	66	2.8	3.1
	70 years or over	7	53	60	2.7	3.0
Sex	Female	8	46	54	2.0	2.1
	Male	9	110	119	Ref	Ref
Race	White	17	146	163	-	-
	Brown	0	4	4	-	-
	Black	0	4	4	-	-
	Information not provided	0	2	2	-	-
Marital status	Married	13	96	109	2.2	2.0
	Single	1	7	8	2.3	2.5
	Widowed	3	30	33	Ref	Ref
	Divorced	0	21	21	Ref	Ref
	Others	0	1	1	Ref	Ref
	Information not provided	0	1	1	-	-
Educational level	Did not complete primary education	8	48	56	2.6	3.0
	Primary education completed	2	22	24	1.5	1.6
	Illiterate	0	6	6	Ref	Ref
	Literate	2	29	31	Ref	Ref
	Did not complete secondary education	0	1	1	Ref	Ref
	Secondary education completed	1	15	16	Ref	Ref
	Higher education completed	0	1	1	Ref	Ref
	Information not provided	4	34	38	-	-
	Diabetic	14	111	125	1.5	1.6
	Not diabetic	3	38	41	Ref	Ref
	Information not provided	0	7	7	-	-
	Hypertensive	15	108	123	2.7	3.0
	Not hypertensive	2	43	45	Ref	Ref
	Information not provided	0	5	5	-	-
	Smoker and ex-smoker	6	73	79	1.7	1.8
	Non-smoker	2	45	47	Ref	Ref
	Information not provided	9	38	47	-	-
	Alcoholic, social drinker, and ex-drinker	2	41	43	0.6	0.6
	No drinking	6	74	80	Ref	Ref
	Information not provided	9	41	50	-	-
Diagnosis	Cardiac disease	1	0	1	11.4	-
	Lung disease	1	2	3	3.8	5.2
	Kidney disease	1	1	2	5.7	10.4
	Infection	1	3	4	2.8	3.5
	Vascular disease	8	28	36	Ref	Ref
	Diabetes	5	81	86	Ref	Ref
	Wound/ulcer	0	20	20	Ref	Ref
	Hansen’s disease	0	5	5	Ref	Ref
	Cancer	0	1	1	Ref	Ref
	Information not provided	0	15	15	-	-
Site	At level of forearm	1	1	2	16.8	32.7
	At level of thigh	11	37	48	7.7	9.7
	At level of leg and lower limbs	2	20	22	3.1	3.3
	At level of foot and tarsal	1	27	28	Ref	Ref
	Hallux	2	48	50	Ref	Ref
	Fingers and toes	0	17	17	Ref	Ref
	Amputation finger by finger	0	3	3	Ref	Ref
	At level of hand and wrist	0	2	2	Ref	Ref
	Amputation of penis	0	1	1	Ref	Ref
Anesthesia	General anesthesia (inhaled and venous)	4	18	22	2.1	2.4
	Spinal anesthesia	13	108	121	Ref	Ref
	Local anesthesia and sedation	0	10	10	Ref	Ref
	Spinal anesthesia and sedation	0	9	9	Ref	Ref
	Local anesthesia	0	5	5	Ref	Ref
	Peridural anesthesia	0	3	3	Ref	Ref
	General anesthesia (venous)	0	2	2	Ref	Ref
	Peridural anesthesia and sedation	0	1	1	Ref	Ref

n indicates the number of occurrences and Ref indicates that these variables were included in the reference group for calculation of the risk ratio and the odds ratio.

 The postoperative mortality rate was 9.8%. Of the amputees, 73.0% were over the age of 60 years and this subset accounted for 88.0% of deaths (OR = 3.0). In the subset of patients over the age of 70 years (35%), 86.9% survived to discharge; however all of those who had embolism and thrombosis of arteries of the lower extremities (1.1%) died. In the subset of patients aged from 60 to 69 years (38.0%), 87.9% survived to discharge; but 66.7% of those who had a diagnosis of unspecified peripheral vascular disease (1.7% of the sample) died. In the subset of patients aged from 40 to 59 years, 93.5% survived to discharge; but among those with type 2 diabetes mellitus with circulatory complications (1.7% of the sample), 66.7% died (Rules 3, 2 and 1 from Chart 1 ). 

 A majority of the amputees were men (68.8%); but the risk and likelihood of death for women were twice those for men (RR = 2.0 and OR = 2.1). Of the female amputees, 85.2% survived to discharge, and all of those with type 2 diabetes mellitus with circulatory complications (1.1%) died ( Chart 1 – Rule 4). 

 White race was present in 94.2% of amputees and in all those who died. In the subset of white patients, 88.3% survived to discharge; however, in the presence of embolism and thrombosis of arteries of the lower extremities or of unspecified peripheral vascular disease (both present in 1.7% of the sample), 66.7% died ( Chart 1 – Rule 7). 

 Married and single patients both had double the likelihood of death than divorced and widowed patients. Married patients accounted for 76.5% of deaths and 63.0% of all patients who had surgery. In the subset of married amputees, 86.2% survived to discharge; but among those with a diagnosis of embolism and thrombosis of arteries of the lower extremities (1.7%), 66.7% died ( Chart 1 – Rule 6). 

 Low educational level was predominant among the amputees: just 1.0% had graduated from higher education and 67.0% had not attended secondary education. Among the 32.0% who had started but not completed primary education, 83.9% survived to discharge, and death was observed in all of those in whom this variable was combined with a diagnosis of embolism and thrombosis of arteries of the lower extremities (1.1%). Among literate amputees (17.8%), 93.5% survived to discharge; and among patients who had completed primary education (13.8%), the discharge rate was 87.5% ( Chart 1 – Rules 10, 8 and 9). Information on profession was missing from 70.0% of records, making a detailed analysis impossible. Notwithstanding, the most common professions were: retired, homemaker, driver, construction worker, and salesperson. 

 Hypertension was present in 71.0% of the amputees and 88.0% of those who died. The likelihood of death was three times greater among hypertensive patients compared with those without hypertension. Of those without hypertension, 93.3% survived to discharge. Diabetes was present in 72.0% of the amputees and in 82.0% of the deaths. In the subset of amputees without diabetes (24%), 87.8% survived to discharge. In the subset of diabetics, 88.1% survived to discharge, but all diabetics with peripheral vascular disease (1.1%) died. Diabetes was present in 86.7% of the hypertensive patients who died (8.7%) (Chart 1 – Rules 11, 14, 16 and 13). 

 With regard to tobacco use, 45.0% of the amputees had used or still used tobacco, which was associated with 35.0% of the deaths. However, in 53.0% of deaths this information was not provided. Among amputees who stated they did not use tobacco (27.0%), 95.7% survived to discharge. Of the 24.0% ex-smokers, 95.2% survived to discharge; whereas 83.8% of the 21.0% of smokers survived to discharge ( Chart 1 – Rules 19, 17 and 18). The order of risks, from higher to lowest, was as follows: smoker, ex-smoker, non-smoker. 

 With regard to alcoholism, 25.0% drank or had drunk alcohol, which was associated with 12.0% of deaths. However, in 53.0% of deaths this information was not provided. Of the 46.0% who stated they did not drink alcohol, 92.4% survived to discharge. However, 66.7% of those who stated they did not drink alcohol and were retired (1.7%) died. All of the amputees who did not drink and had a diagnosis of type 2 diabetes mellitus and peripheral circulatory complications (1.1%) died, and so did all non-drinkers with a diagnosis of peripheral vascular disease (1.1%) ( Chart 1 – Rule 20). 

 In the subset of patients who had more than one amputation (33.9%), 96.6% survived to discharge, and 88.2% of the patients who died had had just one amputation that year. In the subset of patients who had had just one amputation that year (65.5%), 85.1% survived to discharge. Among the 2.9% who had had one amputation and had type 2 diabetes mellitus with circulatory complications, 60.0% died ( Chart 1 – Rules 21, 22 and 25). 

 The analysis of groups of pathologies in the diagnostic hypothesis showed that vascular diseases were the major cause of deaths (47.0%), followed by diabetes (29.4%). However, risk of death was 11.4 times greater in the presence of cardiovascular disease, five times greater with kidney disease (RR = 5.7 and OR = 10.4), three times greater with lung disease (RR = 3.8 and OR = 5.2), and double in the presence of infection (RR = 2.8 and OR = 3.5), when compared with the group of diagnostic hypotheses vascular disease, diabetes, Hansen’s disease, neoplasm, and wound/ulcer. 

 The most common surgeries were hallux (29.0%), thigh (28.0%), and foot and tarsal (16.0%). In the subset of patients who died, 64.0% underwent amputations at the level of the thigh, followed by 12.0% at the hallux and leg, and lower limbs respectively. The risk of death was 16 times higher for surgery at the level of the forearm (RR = 16.8 and OR = 32.7) and seven times higher at the level of the thigh (RR = 7.7 and OR = 9.7), in comparison to surgeries at the level of the foot, tarsal, hallux, fingers and toes, finger by finger, hand and wrist, and penis. Among thigh amputations, 72.9% of the patients survived to discharge, and death was associated with type 2 diabetes mellitus with circulatory complications (1.7%) and with embolism and thrombosis of arteries of the lower extremities (1.1%). After amputation of the leg and lower limb, 90.9% of patients survived to discharge ( Chart 1 – Rules 28 and 26). 

 The type of anesthesia most often used was spinal anesthesia (70.0%), followed by general anesthesia (inhaled and venous) (13.0%). In the subset of patients who died, 76.0% had been given spinal anesthesia, while 10.0% of the patients given spinal anesthesia died. The risk of death was double among patients given general anesthesia (inhaled and venous), compared to all other patients, and general anesthesia was present in 24.0% of deaths. Among deaths after general anesthesia (66.7%), 1.7% had surgery lasting from 0 to 30 minutes. All patients with duration of surgery from 151 to 180 minutes (1.1%) and general anesthesia died. In the subset of amputee patients with general anesthesia, 82.6% survived to discharge ( Chart 1 – Rules 29 and 30). 

 With regard to duration of surgery, in 75.0% of operations with duration from 0 to 30 min the patient survived to discharge; but when this duration of surgery was associated with general anesthesia (inhaled and venous), which was observed in 1.7% of the sample, 66.7% of the patients died. Amputations that resulted in discharge varied as follows: duration 61 to 90 min (86.0% discharge), duration 91 to 120 min (90.2% discharge), and duration 121 to 151 min (85.7% discharge). The greatest survival time was observed after operations with duration from 91 to 120 min, with lower survival associated with longer and shorter durations ( Chart 1 – Rules 31, 32, 33 and 34). 

## DISCUSSION

 The postoperative mortality rate in this study was low (9.8%) in comparison with other studies, which have reported rates of 19.0%. 4 Some studies have reported overall mortality rates at 30 days ranging from 4.0% to 22.0%. 4 ^,^ 5 ^,^ 11 The differences in these values can be explained by underlying differences in health services, surgical decisions, and the patients’ reasons for the amputation decision. 4

 Patients aged over the age of 60 years were three times more likely to die than younger patients, which is a characteristic that has been observed in other studies. 2 ^-^ 5 Fortington et al. found that people over the age of 85 years had a mean survival time of 8.8 months after an amputation, whereas in the subset of younger people, survival was greater than 20 months. 4

 Women had fewer amputations, but died twice as frequently as men. The same scenario was observed in a study by Rolim et al., in which 67.0% of amputations were in men and 33.0% in women, with 30-day mortality rates of 14.0% in men and 15.0% in women; 90-day mortality of 22.0% in men and 26.0% in women; 1-year mortality of 31.0% in men and 36.0% in women; and 5-year mortality of 58.0% in men and 61.0% in women, but this difference was not cited in their study. 5

 Fortington et al. reported that a majority of the amputees were men (60.0%) with a mean age of 72.1 years, while the women (40.0%) had a mean age of 77 years. In that study, 30-day mortality was 28.0% in men and 31.0% in women; 1-year mortality was 49.0% in men and 52.0% in women; and 5-year mortality was 78.0% in men and 80.0% in women. This difference can be explained by the fact that the women had amputations at more advanced ages than the men, since age is an important risk factor. 4

 Mortality was predominantly observed in white patients. This can be explained by the fact that 89.7% of the population of the state of Santa Catarina are white; in addition to the fact that white is the predominant race seen at the study center, because of German immigration. 12 In a study by Lavery et al., the predominant race was Hispanic (78.1%), survival rate was 78.0% 1 year after amputation and 48.0% at 5 years. 13 A similar situation was found in studies with black patients in the Caribbean and in Barbados, at 69.0% and 44.0%, respectively, 14 but these rates are lower than reported for indigenous North-Americans. 15

 The predominant marital status category was married (76.5%), but the chances of death were similar for married (OR = 2.0) and single patients (OR = 2.5), when compared with widowed and divorced amputees. This characteristic was linked to the profile of this population, since other studies have reported that a majority (58.0%) of amputation patients lived alone and just 42.0% lived with a partner. 4 This information is indicative of the different healthcare seeking behaviors in different types of marital status and shows that this attribute should be considered when planning treatment and rehabilitation. 

 The chance of death in hypertensive patients was three times greater than for those who did not have hypertension and the likelihood can increase when associated with diabetes. Studies report that patients with diabetes had greater numbers of minor amputations and twice as many amputations at the transtibial level than transfemoral amputations, in contrast with people who did not have diabetes. 4

 Risk related to tobacco use was observed in the following order, from high to low: smokers, ex-smokers, and non-smokers. This result agrees with a study that stated that 33.0% of patients who had smoked and 25.0% who had never smoked died within 30 days of amputation, that 50.0% of smokers and 46.0% of non-smokers died within 1 year, and that 78.0% of smokers and 77.0% of non-smokers had died within 5 years, 4 confirming that tobacco use is a risk factor for amputation patients. However, for this variable, for alcoholism, and for profession, there were high percentages of incomplete and missing data, making it difficult to conduct an effective analysis. 

 The most common pathologies in post-surgical deaths were vascular disease and diabetes, and the risk was multiplied in the presence of cardiac, renal, and pulmonary disease and infections. Another study stated that the predominant etiology was vascular disease (peripheral arterial occlusive disease – 87.0%) followed by infection (7.0%), and that presence of ischemic heart disease and cerebrovascular disease had a significant impact as a predictive factor of lower survival. 5

 Another study showed that, 45.0% of amputees with cerebrovascular disease, 33.0% with kidney disease, 32.0% with cardiac disease, and 28.0% with chronic pulmonary disease died within 30 days of amputation. The probability of death within 30 days was two to three times greater in people who had cerebrovascular disease than in those who did not. Among people with kidney disease, there was a 3.53 times greater probability of death within 1 year and 5.35 times greater probability within 5 years than in the absence of this morbidity. 4 Analysis of rehospitalization was precluded by the temporal window chosen for data selection. 

 With regard to the level of amputation, the more invasive the amputation, the greater the risk of death. Another study reported survival rates at 30, 90, 365 days and 5 years for patients who underwent minor amputation of 95.0%, 91.0%, 79.0%, and 55.0%, respectively, whereas the figures for patients who underwent major amputations were 82.0%, 70.0%, 62.0%, and 35.0% respectively. 4

 The decision of which type of anesthesia to use for lower limb amputation surgery is a challenge, with preference usually given to local anesthesia and spinal anesthesia; although there is little evidence to support use of a specific type of anesthesia because of the profile of these patients, 16 ^,^ 17 who are at elevated risk of adverse events. 17 In this study, spinal anesthesia was used in 76.0% of amputations that resulted in death; however, even though general anesthesia (inhaled and venous) was only used in 24.0% of amputations that resulted in death, patients given general anesthesia were 2.4 times more likely to die than those who were not. 

 However, Chery et al. 16 reported that local and/or regional anesthesia for patients who underwent major amputation of an extremity were associated with a lower incidence of postoperative pulmonary complications and cardiac arrhythmias, which is probably because this is the type of anesthesia that is more favorable in this situation. Moreira et al. 17 compared the association between spinal anesthesia and general anesthesia in patients who underwent amputations of an extremity, concluding that mortality rates at 30 days and other morbidity rates were similar for both types of anesthesia and that type of anesthesia did not significantly affect morbidity or mortality. 

## CONCLUSIONS

 Determinants associated with postoperative mortality after amputation were age over 60 years (OR = 3.0), female sex (OR = 2.0), white race, hypertension (OR = 3.0), diabetes (OR = 1.6), and smoking (OR = 1.8). 

 The pathologies that most resulted in death were vascular disease (47.0%) and diabetes (29.4%). Increased risk of death was observed in the presence of cardiovascular disease (RR = 11.4), kidney disease (OR = 10.4), lung disease (OR = 5.2), and infection (OR = 3.5). Proximal surgeries were more associated with death than distal surgeries. Anesthesia types most related with death were spinal anesthesia and general anesthesia (inhaled and venous). 

 Data mining made it possible to identify peculiarities of the associations between determinants of death after amputation surgery. For example, exceptions that link variables to different diagnostic hypotheses, increasing the likelihood of death.
